# SR-LMamba: A lane detection model for complex scenes integrating curvelet transform with Mamba architecture

**DOI:** 10.1371/journal.pone.0332873

**Published:** 2025-10-31

**Authors:** Mingliang Chen, Qinhao Jia, Jing Yang, Shuxian Liu

**Affiliations:** School of Computer Science and Technology, Xinjiang University, Urumqi, China; Beijing Institute of Technology, CHINA

## Abstract

Lane detection seeks to accurately identify the position and geometry of lane markings in real-world driving environments. However, existing models often struggle with robustness and accuracy due to insufficient integration of high-level semantic understanding and low-level geometric features. To tackle these limitations, we propose SR-LMamba, a novel lane detection framework built upon the Sketch-and-Refine paradigm of SRLane. At the core of our approach lies LMamba, a lightweight three-stage backbone network that accelerates inference while effectively capturing both geometric structures and sequential patterns through a synergistic combination of curvelet transform and the Mamba architecture. In the Refine stage, we introduce the Criss-Cross Lane Association Module (CLAM), which employs a multi-lane criss-cross attention mechanism to enhance feature interactions and applies polynomial regression to refine lane curve fitting. To further boost performance, we design tailored loss functions—angle loss and criss-cross attention loss—aligned with the model architecture. Experimental results show that SR-LMamba achieves an F1 score of 80.04%, outperforming current state-of-the-art models with similar parameter sizes, and demonstrating superior robustness across four challenging driving scenarios. In addition, we publicly release our code and models at https://github.com/chenml1/SR-LMamba.

## 1. Introduction

Lane detection is a challenging task within the realm of computer vision, with its applications spanning a multitude of domains, including autonomous driving and advanced driver assistance systems (ADAS) [1]. Vehicles equipped with ADAS can detect and sense their surrounding environment, process all the captured information by its equipped sensors, and provide the correct action to the driver rapidly and accurately [2].

Most contemporary lane detection models rely on Convolutional Neural Networks (CNNs) for feature extraction, examples of which include SCNN [3], RESA [4], and UFLD [5]. Nevertheless, the receptive field of CNNs is typically local. Although it is possible to augment the receptive field by employing larger convolutional kernels or increasing the network depth, when compared to Mamba [6], CNNs exhibit relatively weaker capabilities in capturing global context information.

Transformer architectures, on the other hand, possess a global receptive field. Lane detection models based on Transformer [7], such as LSTR [8], leverage the self-attention mechanism to concurrently consider all positional information within the input sequence. This enables the capture of the global structure and context of lanes. However, the quadratic computational complexity of Transformer somewhat restricts the efficiency of extracting detailed features, often resulting in suboptimal performance in practical applications.

Mamba, through the use of the Selective State Space Model (Selective SSM), typically achieves a linear computational complexity. A plethora of computer vision models based on Mamba, such as VMamba [9], MambaVision [10], and Mamba-YOLO [11], attest to the significant research and application potential of Mamba in the field of computer vision.

Mamba’s bidirectional scanning mechanism and positional encoding design endow it with enhanced adaptability to dynamic changes, enabling effective handling of lane continuity and dynamic variations. However, its performance in processing local details may be inadequate.

In other fields, the integration of Mamba with signal processing techniques such as the Fourier transform [12–14]and wavelet transform [15–17] has demonstrated complementarity and efficacy. The Fourier transform, while decomposing signals into global frequency components, forfeits spatial information, rendering it arduous to capture lane edges and curve trajectories. The wavelet transform, although capable of detecting edge information, is contingent upon fixed-direction filters and lacks the ability to adaptively adjust directions to align with the intricate trajectories of lanes.

Consequently, we propose to utilize curvelet transform [18] for signal processing. curvelet transform facilitates hierarchical image analysis through anisotropic basis functions. Scale decomposition captures the global trends at coarse scales and local details at fine scales, while directional decomposition represents curve structures via directional sub-bands. Moreover, curvelet transform compresses lane edge and curve information into a small number of high-energy coefficients. In occluded scenarios, it can assist the model in inferring the complete curve from visible segments. Additionally, its multi-scale decomposition enhances the contrast between lanes and the background, exhibits insensitivity to lighting conditions, and demonstrates robust performance in scenarios with high or low illumination.

**Fig 1:**
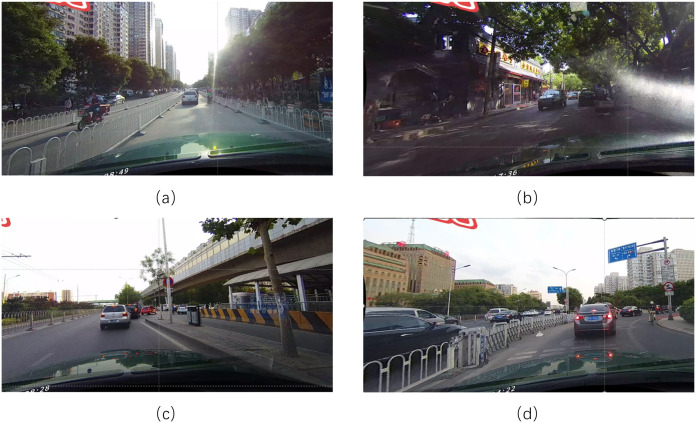
Schematic diagrams of real complex scenes: (a) is a scene with dazzle, (b) is a scene with shadows, (c) is a scene with occlusions, and (d) is a scene with curves.

We endeavor to introduce Mamba into the domain of lane detection. By integrating it with curvelet transform, we design a lightweight backbone network, LMamba, tailored to the lane detection task. Adopting a three-stage network architecture, LMamba not only ensures computational efficiency but also attains a global receptive field, thereby enhancing the model’s practical applicability in complex scenarios. Some complex scenarios are shown in Fig 1.

Current lane detection models often neglect the integration of high-level and low-level semantic features of lanes. High-level semantic features can be used for rough lane localization and distinction from landmarks, while low-level features are suitable for precise localization. Only by fully leveraging both types of features can accurate results be achieved. To make full use of these features, SRLane [19] proposed a new paradigm that combines proposal-based lane detection and keypoint-based lane detection. In the “Sketch” stage, it predicts the local direction map of the lane, initially localizing the lane using low-level features (such as geometric local information). In the “Refine” stage, it understands the high-level semantic information of the lane through a lane association module, better handling the overall structure and complex shapes of the lane. Therefore, we also adopt this paradigm for our research, using LMamba as the backbone network and designing a new lane association module, CLAM, which enhances features through the criss-cross attention mechanism [20]and uses polynomial regression of lane curve parameters to improve the accuracy of lane fitting.

We conduct relevant verifications on the CULane [21] and TuSimple [22] datasets, with particular attention to the performance in the eight complex scenarios of the CULane dataset.

Overall, the contributions of this study are as follows:

- We propose the lane detection backbone network LMamba based on Mamba, which is the first to combine Mamba with the curvelet transform and apply Mamba to the field of lane detection. The three-stage structure design ensures a global receptive field while maintaining lightweight.
- We design the Lane Association Module (CLAM): By leveraging the sequence modeling capability of LMamba and using the criss-cross attention mechanism, we optimize key point localization through polynomial regression, enhancing the model’s fitting accuracy for lanes in complex scenarios.
- According to the model structure, we design and introduce the criss-cross attention loss and angle loss to improve the model’s learning ability.
- Through validation on the CULane and TuSimple datasets and comparison with mainstream CNN-based models, SR-LMamba achieves competitive results, verifying the model’s effectiveness in complex scenarios.

## 2. Related work

This section will introduce the related work from two aspects. One aspect is the current development status of related work in the field of lane line detection. The other aspect is the current application and development of advanced signal processing techniques such as Mamba and curvelet transform in the field of computer vision.

### 2.1. Related work in the field of lane detection

Before the advent of deep learning, traditional computer vision processing methods based on manually fitted features were used for lane detection [23–26]. Generally, color features and edge features were utilized, and lane positioning was achieved through image processing techniques such as the Hough transform and Kalman filter. Currently, lane detection models based on deep learning can be classified into four types: lane detection based on segmentation, lane detection based on anchor points, lane detection based on keypoints, and lane detection based on curve parameters.

#### 2.1.1. Lane detection based on segmentation.

Lane detection based on segmentation regards lane detection as a pixel-level classification problem, that is, determining whether each pixel belongs to a lane. It often has good robustness and accuracy, but it has high requirements for computing resources. Xingang Pan et al. [3] proposed a new convolutional neural network architecture, SCNN (Spatial CNN), for traffic scene understanding. Unlike traditional convolution that processes information layer by layer, SCNN adopts a slice-by-slice convolution method, allowing information to be transmitted between rows and columns of the image, thereby better capturing spatial relationships. They also proved its superiority in lane detection through experiments. However, due to its slow inference speed, it cannot meet the real-time requirements of practical applications. To address the computational time-consuming issue of SCNN, Tu Z et al. [4] proposed RESA, which uses parallel computing. All slices are simultaneously added with the convolution activation results of the previous slices, thus directly operating through indexing without generating slice arrays as in SCNN, improving computational efficiency. Similarly, as an end-to-end lane detection method, LaneNet proposed by Neven, D. et al. [27] regards lane detection as an instance segmentation task, which is achieved through a shared encoder model and two decoder branches (lane segmentation branch and lane embedding branch). The lane segmentation branch is used to distinguish lanes from the background, while the lane embedding branch is used to cluster and distinguish different lanes. However, LaneNet cannot extract features in deep networks due to the lack of processing of receptive fields. To meet the real-time requirements, Zhang L et al. [28] proposed TSA-LNet, which redesigned the encoder-decoder model to generate a lightweight network (Lnet) with fewer parameters, reducing computational load and model complexity. At the same time, it introduced a bidirectional separation attention mechanism (TSA) to enhance the robustness of the model.

#### 2.1.2. Lane detection based on anchor points.

Lane detection based on anchor points mainly involves presetting anchor points in the image and detecting lanes by calculating the offset between the anchor points and the actual lanes. This method has relatively low computational complexity due to the preset anchor points, but the selection of anchor points is often challenging. Line-CNN [29] uses line proposals as references to accurately locate traffic curves and generates multiple predicted lanes for comparison and optimization with the actual lanes. This is the first method to use line anchors for lane detection. LaneATT [30] is a representative of lane detection methods based on anchor points. It uses ResNet [31] as a feature extractor to generate feature maps and then combines an attention mechanism to predict lanes.

Lane detection based on row classification can be regarded as a special case of 2D lane detection based on anchor points, where the anchor points are predefined rows. Ultra Fast Lane Detection (UFLD) [4] effectively reduces the computational load and improves detection speed by selecting row anchor points and utilizing feature points. UFLDv2 [32] treats both rows and columns as anchor points and proposes a hybrid anchor-driven ordinal classification method to achieve ultra-fast deep lane detection.

#### 2.1.3. Lane detection based on keypoints.

Lane detection based on keypoints mainly involves estimating the positions of keypoints and associating keypoints of the same lane, and finally modeling the lane. This method has a relatively high computational efficiency, but its ability to handle occlusions and missing parts is weak. FoloLane [33] regards lane detection as local geometric modeling, determining the position of the lane by predicting keypoints, and gradually building the global lane structure from local information. GANet [34] transforms the lane detection problem into a key point estimation and association problem, determining the shape of the lane by predicting the positions and offsets of keypoints, and aggregating keypoints based on the offsets from the starting point to achieve lane detection. PINet [35] uses key point detection and instance segmentation methods for these keypoints to perform lane detection and clustering, thus enabling end-to-end training and deployment.

#### 2.1.4. Lane detection based on curve parameters.

Lane detection based on curve parameters usually fits the lane by using specific curve models (such as polynomial curves, spline curves, etc.), and determines the position and shape of the lane by solving the relevant parameters of the curve through substituting keypoints. This method is relatively simple in calculation and has relatively high efficiency, but it is highly dependent on the initial parameters of the curve and is sensitive to noise, which may lead to deviations in curve fitting. PolylaneNet [36] directly estimates the shape of the lane through the regression of deep polynomials. Since no prior knowledge is introduced, it can handle various actual roads more flexibly, especially curved lanes. BezierLaneNet, a deep lane detector proposed by Yao Yao et al. [37], detects lanes by regressing Bezier curves, which can effectively model the geometric model of the lane. At the same time, by designing a new feature flipping fusion module based on deformable convolution, it takes advantage of the symmetry of the lane to improve the accuracy of lane detection.

### 2.2. The application of Mamba and Curvelet transforms in computer vision

MamMamba, as an emerging sequence modeling architecture, has demonstrated significant potential in natural language processing and computer vision domains due to its linear computational complexity (O(n)) and efficient long-range dependency modeling capabilities. By employing a selective state space model (SSM) with computation complexity linearly proportional to sequence length, Mamba circumvents the quadratic complexity inherent in Transformer’s self-attention mechanism. Its dynamic input-dependent selective information propagation and forgetting mechanism enables effective long-range dependency capture, addressing the limitation of CNNs in this aspect due to their local receptive fields.

In computer vision, VMamba pioneered the application of Mamba architecture for visual feature extraction through its 2D selective scanning (SS2D) mechanism, achieving a balance between accuracy and speed in tasks such as image classification and object detection by capturing long-range spatial dependencies. MambaVision proposed a hybrid Mamba-Transformer architecture that combines Mamba’s sequential modeling strengths with Transformer’s global interaction advantages, enhancing detail modeling precision in semantic segmentation. Mamba-YOLO integrated Mamba into object detection frameworks, improving real-time detection performance through dynamic state updates for enhanced feature representation of small and dense objects. Variants like FourierMamba [38], WaveMamba [39], and MobileMamba explored Mamba’s integration with signal processing techniques, validating its adaptability in frequency-domain feature modeling.

Considering the specific requirements of lane detection tasks, the ideal signal processing technique must address Mamba’s limitations in capturing local fine-grained features (e.g., edges, textures) while flexibly accommodating two key geometric characteristics: long-range continuity and dynamically changing orientation of lane markings. Fourier transform suffers from spatial information loss during signal decomposition, making it unsuitable for precise lane edge detection. Although Contourlet transform supports multi-directional decomposition, its hierarchical modeling capability at global scales is insufficient to maintain long-range lane continuity. Wavelet and WaveAtom transforms, despite their edge-aware design, rely on fixed directional filters, limiting their adaptability to dynamic lane orientation variations and causing feature distortion in curved or branching road scenarios.

**Fig 2:**
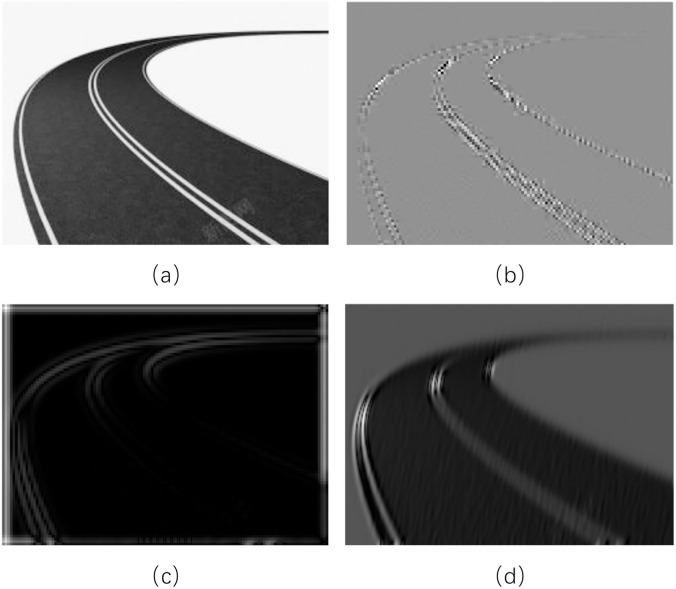
Schematic diagram of signal processing techniques for curve handling: (a) is original image, (b) is the image after contourlet processing, (c) is the image after wavelet processing, and (d) is the image after curvelet processing.

In contrast, curvelet transform efficiently represents dynamically oriented structures like lane markings through scale parameter-controlled feature granularity and direction parameter-adapted curve modeling. Its proven utility in other computer vision applications [40–42] further underscores its value. Therefore, we propose integrating Mamba with curvelet transform for lane detection tasks. Fig 2 illustrates the detailed features extracted by curvelet, wavelet, and contourlet transforms when processing highly curved lanes, demonstrating curvelet transform’s superior capability in capturing lane variations and its suitability for lane detection applications.

## 3. Methods

### 3.1. Overall structure of the model

**Fig 3:**
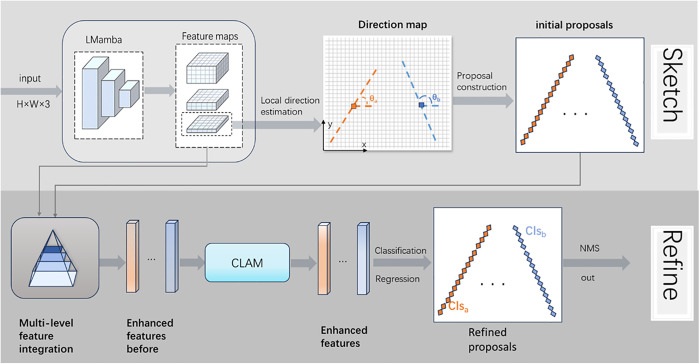
Overall architecture of the proposed SR-LMamba lane detection model. The model follows the “Sketch-and-Refine” paradigm. In the Sketch stage, multi-scale features are extracted by LMamba to generate initial lane proposals based on local direction maps. In the Refine stage, CLAM enhances features using criss-cross attention and refines lane curves via polynomial regression. Arrows indicate the information flow across modules.

Existing lane detection models struggle to efficiently depict lanes. Although SRLane proposed a new paradigm that uses local geometric descriptions to quickly sketch lane shapes and then refines them to improve accuracy, CNN-based backbone networks are often limited by their local receptive fields and have difficulty directly modeling long-range dependencies. Mamba, on the other hand, has a better ability to model long-range dependencies. Therefore, we designed SR-LMamba, which combines Mamba’s long-range dependency modeling capability with the natural adaptability of the curvelet transform to lane targets with clear directionality to design a three-stage backbone network, LMamba. Compared with the four-stage backbone networks [43–45], it offers a faster inference speed. Additionally, we designed CLAM to more effectively capture lane features using a criss-cross attention mechanism and better fit the lanes using polynomial regression.SR-LMamba follows the SRLane detection paradigm, breaking down lane detection into a two-stage process of “Sketch-Refine”, and combines the LMamba backbone network with the Lane Association Module (CLAM) to form an end-to-end detection framework. The overall model is shown in Fig 3, and the specific process is as follows:• Sketch stage: Multi-scale features are extracted through LMamba, and the local direction map of the lanes is predicted by encoding the last feature map, generating sparse keypoint proposals.• Refine stage: Initial proposals and the generated three feature maps are adaptively sampled from the multi-scale feature maps. Then, CLAM enhances the features based on the association information between lanes. After performing Non-Maximum Suppression (NMS) on the refined proposals, the final prediction results are output.

### 3.2. LMamba backbone

**Fig 4:**
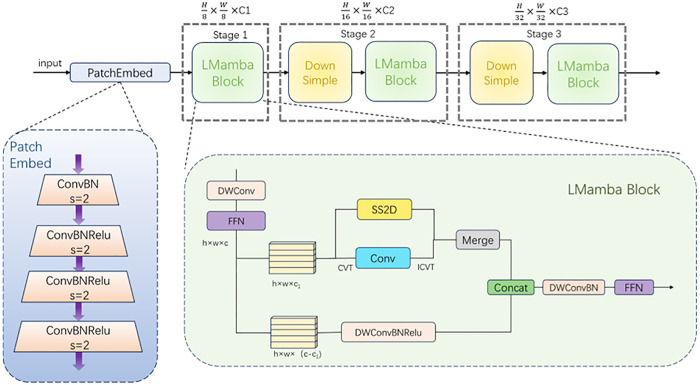
Internal architecture of the LMamba backbone. LMamba consists of three main components: (1) PatchEmbed for initial patch-wise embedding, (2) a dual-branch LMamba Block combining global features via Mamba and curvelet transform with local features via depthwise separable convolution, and (3) downsampling layers to reduce spatial resolution. This design balances global context modeling and local feature preservation.

In this section, we will provide a detailed explanation of the internal structure of LMamba, as shown in Fig 4. Firstly, the input 2D image is divided into patches through PatchEmbed [46], and the feature sequence of each patch is extracted through convolution. Then, through consecutive LMamba Blocks and downsampling, the feature map is generated. Although the three-stage network structure is slightly inferior in performance to the four-stage structure of most CNN-based backbone networks, it can reduce the size of the feature map, thereby reducing the computational load and significantly improving the inference speed.

#### 3.2.1. PatchEmbed.

The function of PatchEmbed is to divide the 2D image into multiple patches, and then embed each patch to output an n-dimensional vector representing this patch. Through a progressive approach, it gradually downsamples by using multiple layers of convolution. The first three convolution steps have a stride of 2 for downsampling, and the last convolution performs channel expansion. Compared with directly dividing, this method retains more edge features and is more in line with the requirements of lane detection tasks.

#### 3.2.2. LMamba Block.

The core requirements of the lane detection task for the model include two aspects: one is the global perception ability, which means being able to capture the long-distance continuity and directionality of the lane; the other is the local detail retention, which involves precisely extracting local features such as lane edges and textures. Based on the requirements of the lane detection task, we designed the LMamba Block.

As shown in Fig 4, the LMamba Block adopts a dual-branch parallel design, consisting of a global branch based on Mamba and curvelet transform, and a local branch based on depthwise separable convolution [47]. The global branch utilizes the characteristic of curvelet transform to adaptively capture the geometric structures in different directions to capture the different directions of the lane, decomposing the image into sub-bands of different scales and directions, and dynamically adjusting the fusion weights with adaptive parameters to achieve complementary weights. The local branch precisely extracts the gradient transformation of lane edges through depthwise separable convolution, distinguishing the lane from the background. At the beginning, we will evenly distribute the fusion weights. Subsequently, during the training process, they will be adjusted according to the backpropagation of the loss function. Finally, the two branches will be fused based on the fusion weights, with the global branch providing directional and long-term dependency information, and the local branch providing edge and detail information, combined to form a complete lane representation.

3.2.2.1. Curvelet transform. The curvelet transform is a multi-scale geometric analysis tool. Compared with traditional wavelet transforms, it has stronger expression capabilities for curves and edges. In the lane detection task, the geometric features of the lane (such as straight lines, curves, and forks) are essentially a collection of structures in different directions. The multi-directional sensitivity of the curvelet transform makes it highly suitable for capturing these features. In the following, we will introduce in detail the mathematical principle, implementation method, and application of the curvelet transform in the LMamba Block in the lane detection task.

The curvelet transform is divided into continuous curvelet transform and discrete curvelet transform [48]. The continuous curvelet transform can be expressed as the inner product of the signal f(x,y) and the curvelet basis function $\phi_{j,\theta ,k}(x,y)$:


$$C(j,\theta ,k)=\left\langle f,\phi_{j,\theta ,k}\right\rangle =\iint \;f(x,y)\overset{―}{\phi_{j,\theta ,k}(x,y)}dxdy$$ (1)

Here, j represents the scale parameter, which controls the size of the curvelet; θ represents the direction parameter, which controls the direction of the curvelet (0 ≤ θ < 2π); k represents the position parameter, which controls the center position of the curvelet; $\phi_{j,\theta ,k}(x,y$ is the curvelet basis function, obtained by scaling, rotating and translating the scale function ϕ(x,y).

In practical applications, for the sake of computational efficiency, the Discrete Curvelet Transform (DCT) is often used. For a two-dimensional image I(m,n), its discrete curvelet transform can be expressed as:


$$\begin{array}{c} C(j,\theta ,p,q)=\sum {\mathrm{\backslash nolimits}}_{m=0}^{M-1}\;\sum {\mathrm{\backslash nolimits}}_{n=0}^{N-1}\;I(m,n)\overset{―}{\phi_{j,\theta ,p,q}(m,n)} \end{array}$$ (2)

Where M × N is the image size; p, q are discrete position parameters, and $\phi_{j,\theta ,p,q}(m,n)$ is the discrete curvelet basis function. LMamba also uses the discrete curvelet transform. When constructing the curvelet basis, it is usually done through frequency domain decomposition and can be expressed as:


$$\hat{C}(j,\theta ,k)=\hat{f}(\xi )\overset{―}{{\hat{\phi }}_{j,\theta ,k}(\xi )}$$ (3)

Here, $\hat{f}(\xi )$ and ${\hat{\phi }}_{j,\theta ,k}(\xi )$ are respectively the Fourier transforms of f(x,y) and $\phi_{j,\theta ,k}(x,y)$.

The key feature of the curvelet transform is the “anisotropic scaling relation”: at scale j, the support region of the curvelet basis function has a length of $2^{-j/2}$ in the direction θ and a length of $2^{-j}$ in the perpendicular direction. This characteristic enables the curvelet to efficiently represent curves and edges, which is in line with the geometric characteristics of the lane. However, the complexity of directly implementing the discrete curvelet transform in the model is very high. Therefore, we use a Gabor filter bank to simulate the directional feature extraction capability of the curvelet transform, so as to achieve the curvelet transform more simply. The Gabor filter is a complex-valued filter that can capture the specific direction and frequency information of an image. Gabor can control its sensitivity to different frequency (coarse and fine) features through the scale parameter, similar to the multi-scale decomposition of curvelet. Compared with other directional filters (such as the Sobel and Scharr filter groups), the Gabor filter group can extract more abundant directional details and is more in line with the curvelet transform.The mathematical expression of the Gabor filter is:


$$g(x,y;\lambda ,\theta ,\psi ,\sigma ,\gamma )=exp\left(-\frac{x^{\prime 2}+\gamma^{\text{2}}y^{\prime 2}}{2\sigma^{\text{2}}}\right)exp\left(i\left(2\pi \frac{x^{\prime }}{\lambda }+\psi \right)\right)$$ (4)

*Among them:*


$$x^{\prime }=xcos\;\theta +ysin\;\theta$$ (5)

$$y^{\prime }=-xsin\;\theta +ycos\;\theta$$ (6)

λ is the wavelength of the sinusoidal factor, θ is the direction of the filter. ψ is the phase shift, σ is the standard deviation of the Gaussian function. γ is the spatial aspect ratio.

In the LMamba model, we use the modulus values of the real and imaginary parts of the Gabor filter as the directional feature extractor:


$$G(x,y;\theta )=\sqrt{{\left[exp\left(-\frac{x^{\prime 2}+\gamma^{\text{2}}y^{\prime 2}}{2\sigma^{\text{2}}}\right)cos\left(2\pi \frac{x^{\prime }}{\lambda }\right)\right]}^{\text{2}}+{\left[exp\left(-\frac{x^{\prime 2}+\gamma^{\text{2}}y^{\prime 2}}{2\sigma^{\text{2}}}\right)sin\left(2\pi \frac{x^{\prime }}{\lambda }\right)\right]}^{\text{2}}}$$ (7)

To better simulate the multi-directional sensitivity of the curvelet transform, we constructed a Gabor filter bank with multiple directions. This filter bank contains 18 filters, each corresponding to an angular subband in the curvelet transform. Through experiments, we verified that the number of directions has a positive impact on accuracy. However, considering the computational cost of the model, we chose 18 directions. The curvelet decomposition operation can be expressed as the convolution of the input feature map X with the decomposition filter D:


Y=CurveletDecompose(X,D)=Conv(X,D) (8)

Among them, Y is the decomposed feature map, which contains 18 directional sub-bands.

The curvelet reconstruction operation can be expressed as the deconvolution of the decomposed feature map Y and the reconstruction filter R:


$$\hat{X}=CurveletReconstruct(Y,R)=ConvTranspose(Y,R)$$ (9)

In the LMamba model, to ensure the stability of the reconstruction, we perform averaging on the reconstruction results:


$$\begin{array}{c} \hat{X}=\frac{\text{1}}{N}\sum {\mathrm{\backslash nolimits}}_{i=1}^{N}\;ConvTranspose(Y_{i},R_{i}) \end{array}$$ (10)

Here, N represents the number of directional sub-bands, $Y_{i}\;$is the i-th directional sub-band, and $R_{i}\;$is the corresponding reconstruction filter.

To capture directional features at different scales, LMamba employs multi-level curvelet transform processing:


$$X_{\text{1}}=CurveletTransform(X_{\text{0}},D_{\text{1}})$$ (11)

$$X_{\text{2}}=CurveletTransform(X_{\text{1}},D_{\text{2}})$$ (12)

$$X_{n}=CurveletTransform(X_{n-1},D_{n})$$ (13)

Here, $X_{\text{0}}$ represents the input feature map, while $X_{\text{1}},X_{\text{2}},\ldots ,X_{n}$ are the outputs of the successive wavelet transforms, and $D_{\text{1}},D_{\text{2}},\ldots ,D_{n}$ are the corresponding decomposition filters at each level.

After each level of wavelet transformation, we perform convolution enhancement and scale reduction on the features:


$$Y_{i}=Conv(X_{i})\times \alpha_{i}$$ (14)

Here, $\alpha_{i\;}$ represents a learnable scale parameter that controls the contribution of each level of features.

Finally, we merge the features of multi-level processing through the curvelet reconstruction:


$$\hat{X}=CurveletReconstruct(Y_{n},R_{n})$$ (15)

3.2.2.2. SS2D(2D-selective-scan). In order to further enhance the modeling ability of the curvelet transform for long-range dependencies, the LMamba model combines the curvelet transform with the simplified sequence model (SS2D).

SS2D is an efficient sequence modeling method that captures long-range dependencies in sequences through a state space model (SSM). The core is to convert the two-dimensional feature map into sequence processing through a four-direction (horizontal, vertical, and two diagonals) scanning strategy. In the LMamba model, the intermediate state calculation of SS2D can be generated by a linear combination of the input features and the historical states, determining the fusion ratio of the current input and historical information. The formula is:


$$y_{t}=\sigma (W_{h}x_{t}+U_{h}s_{t-1})$$ (16)

Here, $x_{t}$ represents the input feature vector at time t (with D being the feature dimension); $s_{t-1}$ is the hidden state at time t-1 (with M being the state dimension); $W_{h}$ is the input feature mapping matrix; $U_{h}$ is the state feedback matrix; σ is the activation function (used in the model as ReLU).

State update equation of the state space:


$$s_{t}=As_{t-1}+By_{t}$$ (17)

A is the state transition matrix (the core parameter for controlling long-term dependencies); B is the intermediate state mapping matrix; $s_{t}$ is the updated hidden state at time t.

3.2.2.3. LMamba local branch. The foundation of LMamba is the State Space Model (SSM), and its core advantage lies in efficiently capturing long-range dependencies through a linear recursive structure (with a time complexity of O(n), where n is the sequence length). However, as a global branch, SSM may not be precise enough when dealing with local short-term dependencies (such as the local spatial relationships of adjacent features, phrase structures). Therefore, LMamba introduces local branches as a supplement, specifically modeling local patterns, complementing the global modeling capability of SSM. However, the local branches, as auxiliary modules, should not excessively increase the computational burden of the model. The local branches constructed through depthwise separable convolution can efficiently capture local features while maintaining low computational overhead. Depthwise separable convolution combines deep convolution (convolution for each input channel separately) and pointwise convolution (1 × 1 convolution for channel fusion), effectively extracting local spatial features. In sequence modeling, this is equivalent to capturing the interaction between adjacent tokens.

When the channel number is C and the convolution kernel size is K, the parameter quantity of the conventional convolution is K^2^ × C × C, while that of the depthwise separable convolution is K^2^ × C + C^2^.

### 3.3. CLAM

The refined head design of the SRLane paradigm is mainly aimed at gradually refining the initially generated lane proposals to enhance the accuracy of lane detection. However, lanes are often composed of multiple consecutive line segments, each of which may have different characteristics and directions. The distribution of lane features in space is often uneven, with some areas having more obvious lane features while others may be affected by factors such as lighting and occlusion, resulting in relatively weaker features. Noisy background information is also a major factor affecting the accuracy of the model. The attention mechanism can automatically focus on these key areas, enhancing the model’s perception and processing ability of important features. At present, there are numerous studies on the attention mechanism in various fields [49], the traditional attention mechanism may have difficulty capturing the local and structured features of lanes, so a criss-cross attention mechanism is needed. Although the traditional criss-cross attention mechanism can capture different sequence relationships, it is not precise and efficient enough in capturing long-distance lane lines and global features. The criss-cross attention mechanism with a cross-shaped design enables each pixel to collect the context information of all pixels on the cross-shaped path. The first recursive operation aggregates information along the horizontal and vertical directions, and this module is repeatedly applied to make each pixel eventually capture the dependency of the entire image. When processing partially occluded lane lines, the model can utilize the context information of the unoccluded part through the criss-cross attention mechanism to accurately determine the direction of the lane line, while the traditional criss-cross attention mechanism may make incorrect judgments due to its inability to effectively aggregate long-distance information. Compared with the multi-head self-attention mechanism, the criss-cross attention mechanism can specifically capture the dependencies in different spatial dimensions (such as the continuous correlation of lane lines between upper and lower pixel rows, or the positional constraints between left and right columns), thereby more accurately modeling the spatial structure of the lane.

**Fig 5:**
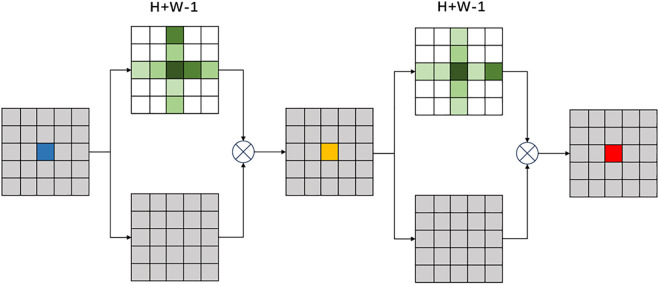
The overall structural of the criss-cross attention mechanism.

The overall structure of the criss-cross attention mechanism is shown in Fig 5. Through two consecutive attentions, it captures information in both row and column directions. This attention mechanism maintains the capture of global information while having a lower computational cost. criss-cross attention has been proven to be superior to non-local, especially in tasks like lane line detection where row and column information is very important.

**Fig 6:**
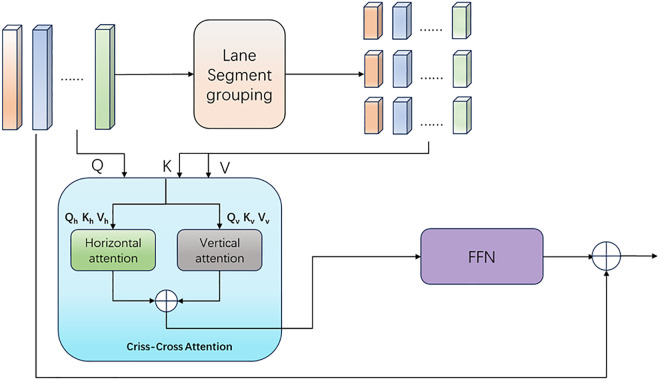
Structure of the Criss-Cross Lane Association Module (CLAM). Features are grouped and separately convolved along horizontal and vertical directions. These directional features are fused using an adaptive gated mechanism to generate the final attention map. Polynomial regression is then applied to generate the lane curves. CLAM improves long-range dependency modeling and fitting accuracy in complex scenarios.

Therefore, as shown in Fig 6, this study adopts a multi-lane criss-cross attention mechanism, groups the features, and performs convolution in the horizontal and vertical directions on different lane groups to obtain the feature representations in these two directions. The results are then fused through an adaptive gated mechanism to obtain the final attention map, and lane fitting is better performed through polynomial regression.

### 3.4. Polynomial regression

The common regression methods may simply predict the coordinates of each point, but they may not be able to fit complex lane line shapes well. Polynomial regression, on the other hand, can fit more complex curves by adjusting the coefficients of the polynomial. For example, for lane lines with a greater degree of curvature, higher-order polynomials can more accurately describe their shapes. Polynomial regression represents the lane line using a small number of coefficients, reducing the number of parameters that need to be learned. This makes the model easier to converge during training and reduces the risk of overfitting, thereby improving the model’s generalization ability.

For a lane line with 72 sampling points, the original regression method may need to predict 72 coordinate values, while polynomial regression only needs to predict a few coefficients of the polynomial.

Suppose we use a d-degree polynomial to represent the relationship between the x-coordinate and y-coordinate of the lane line:


$$\begin{array}{c} x(y)=a_{\text{0}}+a_{\text{1}}y+a_{\text{2}}y^{\text{2}}+\cdots +a_{d}y^{d}=\sum {\mathrm{\backslash nolimits}}_{i=0}^{d}\;a_{i}y^{i} \end{array}$$ (18)

Here, $a_{i}$ represents the coefficients of the polynomial, y is the normalized y-coordinate (usually within the range of [0, 1]), and x(y) is the corresponding x-coordinate. We use a cubic polynomial to represent the relationship between lane coordinates.

### 3.5. Loss function

To better adapt to the model structure and enhance the learning effect of the model, we designed and introduced two loss functions that are strongly related to LMamba and CLAM, namely the criss-cross attention loss and the angle loss.

#### 3.5.1. Angle loss.

Based on the directional characteristics of the curve wave transformation, we designed an angle loss function. The traditional bounding box regression loss only focuses on the positional error of the coordinate points, often ignoring the direction information of the lane. The angle loss can directly optimize the difference between the predicted angle and the real angle, making the model pay more attention to the directional characteristics of the lane. The angle information can not only more effectively describe the curve changes of the lane, but also provide additional constraints, reducing detection ambiguity at intersections or in areas with dense lanes.

The mathematical formula of the angle loss can be expressed as:


$$\begin{array}{c} {ℒ}_{Ang}=\sum {\mathrm{\backslash nolimits}}_{i=1}^{N}\;w_{i}\cdot \frac{\sum_{j\in valid}\;\;|{pred}_{j}-{target}_{j}|}{\sum_{j\in valid}\;\;1+\epsilon } \end{array}$$ (19)

Here, N represents the number of samples in the batch, $w_{i\;}$ is the weight of the i-th sample, ${pred}_{j}$ and ${target}_{j}$ are the predicted angle and the true angle respectively, and “valid” indicates the mask of valid samples. ∊ is a small constant (1e-4) to prevent the denominator from being zero.

#### 3.5.2. Criss-cross attention loss.

To enhance the model’s accuracy, we designed the criss-cross attention loss, which consists of three parts: the attention losses in the horizontal and vertical directions, and the continuity loss. The losses in the horizontal and vertical directions can be expressed as:


$${ℒ}_{h}=NLLLoss(log\;(A_{h}),I_{h},ignorebackslash\_index=255)$$ (20)

$${ℒ}_{v}=NLLLoss(log\;(A_{v}),I_{v},ignore\backslash \_index=255)$$ (21)

$A_{h}$ and $A_{v}$ respectively represent the attention weights (predicted probability distributions) in the horizontal and vertical directions. $I_{h}$ and $I_{v}$ respectively represent the minimum distance indices in the horizontal and vertical directions, which point to the predicted lane points closest to the target lane. “NLLLoss”: negative log-likelihood loss function, used to evaluate the difference between the prediction and the target. Set 255 as the ignored index to prevent invalid points or regions from being considered during loss calculation.

The continuity loss is calculated by the second-order difference between the predicted lane points to ensure the continuity of the lane, making the changes between adjacent lane points smooth. The calculation formula for the continuity loss of the predicted lane P is as follows:


$$\begin{array}{c} {ℒ}_{c}=\frac{\text{1}}{n_{prior}\times (72-2)}\sum {\mathrm{\backslash nolimits}}_{i=1}^{n_{prior}}\;\sum {\mathrm{\backslash nolimits}}_{j=1}^{72-2}\;|\Delta^{\text{2}}P_{ij}| \end{array}$$ (22)

The expression $\Delta^{\text{2}}P_{ij}=P_{i,j+2}-2P_{i,j+1}+P_{i,j}$ represents the second-order difference.

The horizontal and vertical losses are weighted and summed with the continuity loss to obtain the criss-cross attention loss:


$${ℒ}_{c-attn}=\lambda_{h}{ℒ}_{h}+\lambda_{v}{ℒ}_{v}+\lambda_{c}{ℒ}_{c}$$ (23)

#### 3.5.3. Total loss.

In addition to the losses mentioned above, our model also has focal loss for classification and IOU loss for regression. The total loss function is as follows, where ω is a hyperparameter:


$${ℒ}_{Total}={\omega_{cls}ℒ}_{cls}{{+\omega }_{reg}ℒ}_{reg}+{\omega_{c-attn}ℒ}_{c-attn}{{+\omega }_{Ang}ℒ}_{Ang}$$ (24)

## 4. Experimental Results and Analysis

### 4.1. Dataset and evaluation metrics

This study used two large public lane detection datasets, TuSimple and CULane, for model training and validation. The TuSimple dataset contains approximately 7,000 one-second-long video clips, each consisting of 20 frames of images, but only the last frame was labeled with detailed lane information. The dataset is divided into a training set and a test set, with 3,626 and 2,782 video clips respectively, covering different weather conditions (sunny, cloudy, rainy), time periods, lane numbers, and traffic conditions.

The CULane dataset was collected by installing cameras on six vehicles driven by different drivers in Beijing at different times. A total of over 55 hours of video was collected, from which 133,235 frames of images were extracted. The dataset is divided into a training set, a validation set, and a test set, with 88,880, 9,675, and 34,680 images respectively. The CULane dataset includes different scenarios such as urban and rural highways.

This study selected the F1 score of CULane and the accuracy of TuSimple as evaluation metrics. In addition to accuracy on the TuSimple dataset, we also reported the F1, FP, and FN ratios.


$$Accuracy=\frac{TP+TN}{TP+FP+TN+FN}$$ (25)

$$F1=2\times \frac{Precision\times Recall}{Precision+Recall}$$ (26)

### 4.2. Implementation details

The LMamba backbone network is used for feature extraction. All input images are adjusted to 320 × 800, and data augmentation methods such as rotation, translation, and flipping will be applied. Set batchsize to 40, default lane grouping to 6, maximum number of lanes to 5. All models are trained using the AdamW optimizer, with an initial learning rate of 5e-4, weight decay set to 0.05, and trained for 50 epochs. A cosine annealing learning rate scheduling strategy is adopted, and a warm-up stage of 1200 iterations is added. To improve training stability, mixed precision training (16-bit) is used. The CUDA version is 11.8, and the Pytorch version is 2.1.2.4.3. Comparison with other Baselines

We mainly compare with the current state-of-the-art models. Thanks to the unique design of Mamba, the parameter quantity of LMamba is only 21M, which is basically equivalent to ResNet34. When making comparisons, we chose models with different variants of ResNet as the backbone network for comparison. As shown in Table 1, the comparison results of this model with other models on the CULane dataset are presented. It can be seen that the overall performance is very excellent. Compared with the model using ResNet34 as the backbone network, the performance has been comprehensively improved. Compared with the CLRNet using ResNet101, it is slightly inferior. However, it achieved the best results in four scenarios: glare, shadow, no lane marking, and curve. Especially in the glare scenario, it reached 77.34%. Compared with the previous highest score CLRNet, it improved by 3.37%. This is mainly because the curvelet transform is less dependent on lighting and has stronger adaptability to curves. This is also beneficial for the no lane marking scenario. At the same time, we also reported that although FPS and SR-LMamba are not the best, compared to other models, they still have certain competitiveness in terms of real-time processing.

**Table 1:** The comparison results with other baselines on CULane.

Method	Backbone	Normal	Crowd	Dazzle	Shadow	No line	Arrow	Curve	Cross↓	Night	Total	FPS
SCNN	VGG16	90.60	67.90	58.50	66.90	43.40	84.10	64.40	1990	66.10	71.60	7.5
RESA	ResNet34	91.90	72.40	66.50	72.00	46.30	88.10	68.60	1896	69.80	74.50	45.5
RESA	ResNet50	92.10	73.10	69.20	72.80	47.70	88.30	70.30	1503	69.90	75.30	35.7
UFLD	ResNet34	90.70	70.20	59.50	69.30	44.40	85.70	69.50	2037	66.70	72.30	170
UFLDV2	ResNet34	92.50	74.80	65.50	75.50	49.20	88.80	70.10	1910	70.80	76.00	91
LaneATT	ResNet34	92.14	75.03	66.47	76.31	49.46	88.29	67.75	1360	70.72	77.68	129
LaneATT	ResNet152	91.74	73.16	69.47	78.15	50.39	86.38	64.02	1264	70.81	76.02	20
SCNet [50]	ResNet54	92.07	75.41	67.75	74.31	50.90	87.97	69.65	1375	72.69	77.27	92
CondLane [51]	ResNet34	93.38	78.33	73.71	79.66	53.14	90.25	71.56	1321	75.11	78.74	128
CondLane	ResNet101	93.47	77.44	70.93	80.91	54.13	90.16	75.21	1201	74.80	79.48	47
CLRNet [52]	ResNet34	93.49	75.06	74.57	79.92	54.01	90.59	72.77	1216	75.02	79.61	103
CLRNet	ResNet101	**93.85**	**78.78**	72.49	82.33	54.50	89.73	75.42	1290	**75.37**	**80.07**	46
SRLane	ResNet18	93.45	77.74	71.35	79.12	51.98	90.31	74.67	1365	74.70	78.90	**221**
SRLane	ResNet34	93.52	77.96	73.84	81.90	55.35	89.50	75.27	1412	74.86	79.63	146
SR-LMamba	LMamba	93.77	78.66	**77.34**	**83.41**	**56.29**	90.36	**76.56**	1422	75.24	80.04	137

Table 2 shows the comparison results of the model on the TuSimple dataset, mainly focusing on its accuracy and F1 score. However, compared with other models, the performance of this model is slightly lower, even with a minor decrease. This is because the curvelet transform is more sensitive to directionality and curve construction, while the lanes in this dataset are often straight, which prevents the model from fully leveraging its advantages.

**Table 2:** The comparison results with other baselines on TuSimple.

Method	Backbone	F1(%)↑	Acc(%)↑	FP(%)↓	FN(%)↓
SCNN	ResNet18	95.97	96.53	6.17	**1.80**
PolyLaneNet	ResNet34	93.14	93.36	9.42	9.33
RESA	ResNet50	96.93	96.82	3.63	2.48
UFLD	ResNet34	88.02	95.86	18.91	3.75
UFLDV2	ResNet34	96.22	95.56	3.18	4.37
LaneATT	ResNet34	96.77	95.63	3.53	2.92
LaneATT	ResNet152	96.06	96.10	5.64	2.17
CondLane	ResNet34	96.98	95.37	2.20	3.82
CondLane	ResNet101	97.24	96.54	**2.01**	3.50
CLRNet	ResNet34	**97.82**	96.84	2.28	1.92
CLRNet	ResNet101	97.62	96.83	2.37	2.38
SRLane	ResNet18	97.66	**96.85**	2.80	1.85
SRLane	ResNet34	97.58	96.81	2.92	1.93
SR-LMamba	LMamba	97.62	96.84	2.71	**1.80**

### 4.4. Ablation experiment

To verify the effectiveness of our design, we used ResNet34 and LMamba as the backbone on CULane respectively, gradually adding the CLAM module. The results are shown in Table 3. It can be seen that LMamba, as the backbone for lane detection, has achieved good results in complex scenarios due to its use of the curvelet transform, which is insensitive to lighting and has directionality. In scenarios with strong lighting noise and curved scenes, it has performed well. To better demonstrate the superiority of the combination of Mamba and curvelet transform in the lane detection scenario, we also replaced the original ResNet of the model with some backbone networks based on Mamba and compared the results. It was found that using VMamba as the backbone network was not effective, MobileMamba, as a backbone network combining wavelet transformation with the Mamba backbone architecture, although it performs well, still falls short of LMamba.. The criss-cross attention mechanism adopted by CLAM is more in line with the requirements of the lane detection task and has played a role in multiple complex scenarios. Due to the strong correlation between the added loss function and the modules, since we did not conduct separate ablation experiments on the loss function, but instead added angle loss when conducting ablation experiments on LMamba, and added criss-cross attention loss when conducting ablation experiments on CLAM, the results proved that using LMamba as the backbone network was effective.

**Table 3:** The ablation experiment results of the module effect on CULane.

Model	Normal	Crowd	Dazzle	Shadow	No line	Arrow	Curve	Cross↓	Night	Total
ResNet34	93.52	77.96	73.84	81.90	55.35	89.50	75.27	1412	74.86	79.63
ResNet34 + CLAM	93.48	78.46	74.35	81.87	55.36	89.68	75.33	1424	75.16	79.74
VMamba	70.31	46.61	40.37	35.52	30.75	70.04	50.25	2687	50.36	49.71
MobileMamba	89.49	72.18	65.09	71.47	47.74	84.01	59.67	2041	66.57	70.01
LMamba	93.53	78.89	76.53	82.54	55.61	90.13	75.80	1436	74.95	79.83
LMamba+CLAM	93.77	78.66	77.34	83.41	56.29	90.36	76.56	1422	75.24	80.04

At the same time, we also conducted ablation experiments on the angle loss and the cross-attention loss in the model. The results are shown in Table 4, which verified the effectiveness of the loss function design.

**Table 4:** Loss function ablation experiment results.

Angle loss	Criss-cross attention loss	F1
✔		79.92
	✔	79.34
✔	✔	80.04

### 4.4. Visualization results

**Fig 7:**
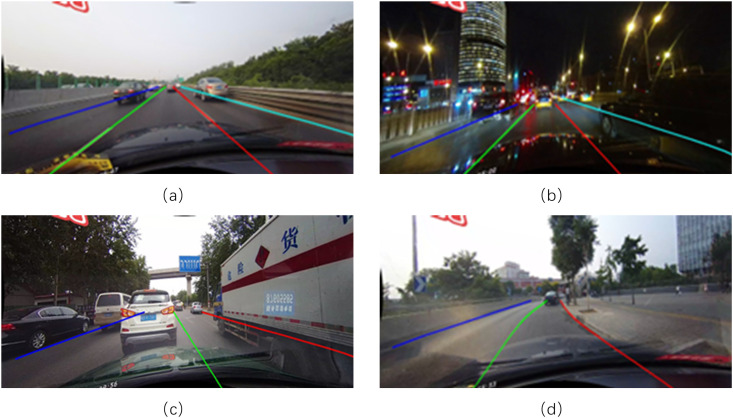
Visualization of SR-LMamba Performance Across Diverse Scenarios. The images used in this figure are generated using data from the CULane dataset [21].

Fig 7 presents the visualization results of our designed SR-LMamba model in some scenarios. The results show that our model can maintain good performance in various complex scenarios that actually exist.

## 5. Conclusions

In this paper, we propose a new lane detection model named SR-LMamba, and design a new LMamba backbone network. Through a three-stage, dual-branch structure, we utilize the curvelet transform and Mamba effectively to extract lane features. At the same time, we design the CLAM module to enhance the features using the criss-cross attention mechanism. Additionally, we have specially designed a new loss function to better fit the structure of our model. Our model is evaluated on two datasets, CULane and TuSimple. The experiments prove that our model has good performance in complex scenarios.

Although we employed some lightweighting methods, the number of parameters in the model is still high. In the future, we will also adopt more lightweighting approaches to improve the inference speed of the model, so that it can be used in practical applications. We also believe that the LMamba backbone network will have its applicability for more directional scenarios.

## References

[1] Hillel RB, Lerner D, Levi D, Raz G. Recent progress in road and lane detection: a survey. Mach Vis Appl. 2014;25(3):727–45.

[2] Assemlali H, Bouhsissin S, Sael N. Deep learning-driven CNN model for detection and classification of dynamic obstacles. Green Energy and Intelligent Transportation. 2025;100334. doi: 10.1016/j.geits.2025.100334

[3] Pan X, Shi J, Luo P, Wang X, Tang X. Spatial as deep: spatial CNN for traffic scene understanding. AAAI. 2018;32(1). doi: 10.1609/aaai.v32i1.12301

[4] Zheng T, Fang H, Zhang Y, Tang W, Yang Z, Liu H, et al. RESA: recurrent feature-shift aggregator for lane detection. AAAI. 2021;35(4):3547–54. doi: 10.1609/aaai.v35i4.16469

[5] Qin Z, Zhang P, Li X. Ultra fast deep lane detection with hybrid anchor driven ordinal classification. IEEE Trans Pattern Anal Mach Intell. 2024;46(5):2555–68. doi: 10.1109/TPAMI.2022.3182097 35696463

[6] Gu A, Dao T. Mamba: linear-time sequence modeling with selective state spaces. ArXiv. 2023. doi: abs/2312.00752

[7] Vaswani A, Shazeer N, Parmar N, et al. Attention is all you need. arXiv. 2017. https://arxiv.org/abs/1706.03762

[8] Wang Z, Guo J, Hu Z, Zhang H, Zhang J, Pu J. Lane transformer: a high-efficiency trajectory prediction model. IEEE Open J Intell Transp Syst. 2023;4:2–13. doi: 10.1109/ojits.2023.3233952

[9] Liu Y, Tian Y, Zhao Y. Vmamba: visual state space model. Advances in Neural Information Processing Systems. 2024;37:103031–63.

[10] Hatamizadeh A, Kautz J. MambaVision: A Hybrid Mamba-Transformer Vision Backbone. Proceedings of the Computer Vision and Pattern Recognition. 2025;25261–70. 10.1109/cvpr52734.2025.02352

[11] Wang Z, Li C, Xu H. Mamba YOLO: SSMs-based YOLO for object detection. arXiv preprint. 2024. doi: 10.48550/arXiv.2406.05835

[12] Li D, Liu Y, Fu X. Fouriermamba: Fourier learning integration with state space models for image deraining. arXiv preprint. 2024. doi: arXiv:2405.19450

[13] Chang A, Zeng J, Huang R. EM-Net: Efficient Channel and Frequency Learning with Mamba for 3D Medical Image Segmentation. International Conference on Medical Image Computing and Computer-Assisted Intervention. Cham: Springer Nature Switzerland; 2024;266–75.

[14] Xiao Y, Yuan Q, Jiang K, et al. Frequency-assisted Mamba for remote sensing image super-resolution. arXiv preprint arXiv:2405.04964. 2024.

[15] Huang Y, Miyazaki T, Liu X. IRSRMamba: Infrared Image Super-Resolution via Mamba-based Wavelet Transform Feature Modulation Model. arXiv preprint. 2024. doi: arXiv:2405.09873

[16] Ahmad M, Usama M, Mazzara M, Distefano S. WaveMamba: Spatial-Spectral Wavelet Mamba for Hyperspectral Image Classification. IEEE Geosci Remote Sensing Lett. 2025;22:1–5. doi: 10.1109/lgrs.2024.3506034

[17] He H, Zhang J, Cai Y, Chen H, Hu X, Gan Z, et al. MobileMamba: Lightweight Multi-Receptive Visual Mamba Network. In: 2025 IEEE/CVF Conference on Computer Vision and Pattern Recognition (CVPR). 2025;4497–507. 10.1109/cvpr52734.2025.00424

[18] Donoho DL, Duncan MR. Digital curvelet transform: strategy, implementation, and experiments. In: Proceedings of SPIE - The International Society for Optical Engineering. 2000. 12–30.

[19] Chen C, Liu J, Zhou C, Tang J, Wu G. Sketch and refine: towards fast and accurate lane detection. AAAI. 2024;38(2):1001–9. doi: 10.1609/aaai.v38i2.27860

[20] Huang Z, Wang X, Huang L, Huang C, Wei Y, Liu W. CCNet: Criss-Cross Attention for Semantic Segmentation. In: 2019 IEEE/CVF International Conference on Computer Vision (ICCV). 2019. 603–12. 10.1109/iccv.2019.00069

[21] CULane Dataset. https://xingangpan.github.io/projects/CULane.html. 2024.

[22] Zhou K, Liu S. TuSimple lane detection challenge. In: 2017 IEEE/CVF Conference on Computer Vision and Pattern Recognition Workshops (CVPRW). 2017. https://github.com/TuSimple/TuSimple-benchmark/tree/master/doc/lane_detection

[23] Borkar A, Hayes M, Smith MT. Robust lane detection and tracking with ransac and Kalman filter. In: 2009 16th IEEE International Conference on Image Processing (ICIP). 2009. 3261–4. 10.1109/icip.2009.5413980

[24] Deng G, Wu Y. Double Lane Line Edge Detection Method Based on Constraint Conditions Hough Transform. In: 2018 17th International Symposium on Distributed Computing and Applications for Business Engineering and Science (DCABES). 2018. 107–10. 10.1109/dcabes.2018.00037

[25] Yoo JH, Lee S-W, Park S-K, Kim DH. A Robust Lane Detection Method Based on Vanishing Point Estimation Using the Relevance of Line Segments. IEEE Trans Intell Transport Syst. 2017;18(12):3254–66. doi: 10.1109/tits.2017.2679222

[26] Ma H, Ma Y, Jiao J, Bhutta MUM, Bocus MJ, Wang L, et al. Multiple Lane Detection Algorithm Based on Optimised Dense Disparity Map Estimation. 2018 IEEE International Conference on Imaging Systems and Techniques (IST). 2018. 1–5. 10.1109/ist.2018.8577122

[27] Neven D, Brabandere BD, Georgoulis S, Proesmans M, Gool LV. Towards End-to-End Lane Detection: an Instance Segmentation Approach. 2018 IEEE Intelligent Vehicles Symposium (IV). 2018;286–91. 10.1109/ivs.2018.8500547

[28] Zhang L, Jiang F, Yang J, Kong B, Hussain A. A real‐time lane detection network using two‐directional separation attention. Computer aided Civil Eng. 2023;39(1):86–101. doi: 10.1111/mice.13051

[29] Li X, Li J, Hu X, Yang J. Line-CNN: end-to-end traffic line detection with line proposal unit. IEEE Trans Intell Transport Syst. 2020;21(1):248–58. doi: 10.1109/tits.2019.2890870

[30] Tabelini L, Berriel R, Paixao TM, Badue C, De Souza AF, Oliveira-Santos T. Keep your eyes on the lane: Real-time attention-guided lane detection. In: IEEE/CVF Conference on Computer Vision and Pattern Recognition (CVPR). Piscataway, NJ: IEEE; 2021;294–302.

[31] He K, Zhang X, Ren S, Sun J. Deep Residual Learning for Image Recognition. In: Proceedings of the IEEE Conference on Computer Vision and Pattern Recognition (CVPR). 2016.

[32] Zhang H, Song J, Hu J. Lane Line Detection Algorithm Based on Global Attention and Reconstruction of Spatial Pyramid Pooling. 2024 4th International Conference on Electronic Information Engineering and Computer (EIECT). IEEE. 2024; 373–7.

[33] Focus on Local: Detecting Lane Marker from Bottom Up via Keypoint. 2021 IEEE/CVF Conference on computer Vision and Pattern Recognition (CVPR). 2021;14117–25.

[34] Wang Z, Zhang Y, Liu X. A keypoint-based global association network for lane detection. In: Proceedings of the IEEE/CVF Conference on Computer Vision and Pattern Recognition (CVPR). 2022.

[35] Ko Y, Lee Y, Azam S, Munir F, Jeon M, Pedrycz W. Key Points Estimation and Point Instance Segmentation Approach for Lane Detection. IEEE Trans Intell Transport Syst. 2022;23(7):8949–58. doi: 10.1109/tits.2021.3088488

[36] Tabelini L, Berriel R, Paixao TM, Badue C, De Souza AF, Oliveira-Santos T. PolyLaneNet: Lane Estimation via Deep Polynomial Regression. In: 2020 25th International Conference on Pattern Recognition (ICPR). 2021;6150–6. 10.1109/icpr48806.2021.9412265

[37] Yao Y, Wu Z, Xu Z. Rethinking Efficient Lane Detection via Curve Modeling. In: Proceedings of the IEEE/CVF Conference on Computer Vision and Pattern Recognition (CVPR). 2022.

[38] Li D, Liu Y, Fu X. Fouriermamba: Fourier learning integration with state space models for image deraining. arxiv preprint. 2024. doi: arxiv:2405.19450

[39] Zou W Dr, Gao H Prof, Yang W Dr, Liu T Dr. Wave-Mamba: Wavelet State Space Model for Ultra-High-Definition Low-Light Image Enhancement. In: Proceedings of the 32nd ACM International Conference on Multimedia. 2024;1534–43. 10.1145/3664647.3681580

[40] Ghislain F, Beaudelaire ST, Daniel T. An accurate unsupervised extraction of retinal vasculature using curvelet transform and classical morphological operators. Comput Biol Med. 2024;178:108801. doi: 10.1016/j.compbiomed.2024.108801 38917533

[41] Yulong D, Ke D, Chunsheng O, Yingshe L, Yu T, Jianyi F, et al. Wavelets and curvelets transform for image denoising to damage identification of thin plate. Results Eng. 2023;17:100837. doi: 10.1016/j.rineng.2022.100837

[42] Zhao X, Jin S, Bian G, Cui Y, Wang J, Zhou B. A curvelet-transform-based image fusion method incorporating side-scan sonar image features. JMSE. 2023;11(7):1291. doi: 10.3390/jmse11071291

[43] Chen J, Kao SH, He H, Zhuo W, Wen S, Lee CH, et al. Run, don’t walk: chasing higher flops for faster neural networks. CVPR. 2023.

[44] Li Y, Yuan G, Wen Y, Hu J, Evangelidis G, Tulyakov S, et al. Efficientformer: Vision transformers at mobilenet speed. NeurIPS. 2022.

[45] Yu W, Luo M, Zhou P, Si C, Zhou Y, Wang X, et al. Metaformer is actually what you need for vision. CVPR. 2022.

[46] Liu Z, Lin Y, Cao Y. Swin transformer: hierarchical vision transformer using shifted windows. In: Proceedings of the IEEE/CVF International Conference on Computer Vision. 2021;10012–22.

[47] Howard AG. Mobilenets: Efficient convolutional neural networks for mobile vision applications. arXiv preprint. 2017. doi: 10.48550/arXiv:1704.04861

[48] Candès EJ, Donoho DL. New tight frames of curvelets and optimal representations of objects with piecewise C2 singularities. Comm Pure Appl Math. 2003;57(2):219–66. doi: 10.1002/cpa.10116

[49] Khan H, Ullah Khan S, Ullah W, Wook Baik S. Optimal features driven hybrid attention network for effective video summarization. Engineering Applicat Artificial Intelligence. 2025;158:111211. doi: 10.1016/j.engappai.2025.111211

[50] Su J, Chen C, Zhang K, Luo J, Wei X, Wei X. Structure guided lane detection. 2021. https://arxiv.org/abs/2105.05403

[51] Liu L, Chen X, Zhu S, Tan P. Condlanenet: A top-to-down lane detection framework based on conditional convolution. 2021. https://arxiv.org/abs/2105.05003

[52] Zheng T, et al. CLRNet: Cross layer refinement network for lane detection. In: Proceedings of the IEEE/CVF Conference on Computer Vision and Pattern Recognition, 2022. 898–907.

